# From High-Throughput Microarray-Based Screening to Clinical Application: The Development of a Second Generation Multigene Test for Breast Cancer Prognosis

**DOI:** 10.3390/microarrays2030243

**Published:** 2013-08-29

**Authors:** Jan C. Brase, Ralf Kronenwett, Christoph Petry, Carsten Denkert, Marcus Schmidt

**Affiliations:** 1 Sividon Diagnostics GmbH, Nattermannallee 1, 50829, Cologne, Germany; E-Mails: Kronenwett@sividon.com (R.K.); petry@sividon.com (C.P.); 2 Institute of Pathology, Charité University Medicine Berlin, Charitéplatz 1, 10117 Berlin, Germany; E-Mail: carsten.denkert@charite.de; 3 Department of Gynecology and Obstetrics, University of Mainz, Langenbeckstr. 1, 55131 Mainz, Germany; E-Mail: Marcus.Schmidt@unimedizin-mainz.de

**Keywords:** breast cancer, EndoPredict, multigene test, endocrine therapy

## Abstract

Several multigene tests have been developed for breast cancer patients to predict the individual risk of recurrence. Most of the first generation tests rely on proliferation-associated genes and are commonly carried out in central reference laboratories. Here, we describe the development of a second generation multigene assay, the EndoPredict test, a prognostic multigene expression test for estrogen receptor (ER) positive, human epidermal growth factor receptor (HER2) negative (ER+/HER2−) breast cancer patients. The EndoPredict gene signature was initially established in a large high-throughput microarray-based screening study. The key steps for biomarker identification are discussed in detail, in comparison to the establishment of other multigene signatures. After biomarker selection, genes and algorithms were transferred to a diagnostic platform (reverse transcription quantitative PCR (RT-qPCR)) to allow for assaying formalin-fixed, paraffin-embedded (FFPE) samples. A comprehensive analytical validation was performed and a prospective proficiency testing study with seven pathological laboratories finally proved that EndoPredict can be reliably used in the decentralized setting. Three independent large clinical validation studies (n = 2,257) demonstrated that EndoPredict offers independent prognostic information beyond current clinicopathological parameters and clinical guidelines. The review article summarizes several important steps that should be considered for the development process of a second generation multigene test and offers a means for transferring a microarray signature from the research laboratory to clinical practice.

## 1. Background—Establishment and Clinical Validation of First Generation Multigene Tests for Breast Cancer Patients

Breast cancer is the most common cancer in women worldwide, with a high number of cancer-related fatalities [1]. The decision on how to best treat a breast cancer patient is generally made based on clinical guidelines. They primarily use standard clinicopathological parameters, like age, tumor size, nodal status, grading and hormone receptor status, to define individual prognosis and to categorize patients into clinical stages. Clinical variables have also been integrated in clinical prediction models, such as Adjuvant!Online [2] and the Nottingham Prognostic Index [3,4]. However, guidelines and clinical prediction models rarely provide unambiguous treatment recommendation and do not fully capture the clinical course of the disease. This is because breast cancer is biologically heterogeneous, and molecular differences can lead to a differing outcome, even among tumors with similar clinical characteristics. 

One of the most pressing clinical questions in the management of estrogen receptor (ER) positive, human epidermal growth factor receptor (HER2) negative patients not satisfactorily addressed by current guidelines is whether to limit systemic treatment to just endocrine therapy or to employ adjuvant chemotherapy. While chemotherapy has been shown to provide an overall improved therapy outcome, this benefit is known to be limited to a subgroup of the patients. Still, in most countries, the established clinical practice is to treat the vast majority of patients with an anthracycline- and taxane-containing adjuvant chemotherapy, resulting in considerable overtreatment. To address this clinical need, the St. Gallen expert board has recommended since 2009 integrating molecular data into prognostic and predictive models and using validated multigene tests to assist in deciding whether to add chemotherapy to endocrine therapy [5].

The application of gene expression profiling with the use of microarrays has allowed for measuring thousands of mRNAs in parallel to identify markers that reflect molecular heterogeneity. In 2000, Perou *et al.* identified by unsupervised hierarchical cluster analysis that breast cancer can be subdivided into at least four molecularly distinct subgroups using an intrinsic gene signature [6]. Later, the molecularly distinct subtypes were repeatedly found to be associated with prognosis and response to chemotherapy treatment [7,8,9,10]. 

Over the past decade, many gene expression signatures have been established, but only a few of these have progressed to commercial availability. Investigators from the Netherlands Cancer Institute (NKI) in Amsterdam developed the first prognostic gene signature (Mammaprint, Agendia) for breast cancer patients in 2002 [11]. The signature is based on the measurement of 70 genes and was established in a retrospective series of 78 tumor samples using global gene expression profiles. A statistical “top-down” approach was applied to determine the most relevant genes that were associated with early recurrence in untreated node-negative breast cancer patients [11]. The performance of the 70-gene signature was subsequently validated in a consecutive series of 295 node-negative and node-positive breast cancer patients from the same institution [12]. However, in this validation study, 46% of all patients received adjuvant endocrine or chemotherapy therapy, and the samples had been partially used to establish the Mammaprint assay. Therefore, the validation study raised some concerns about whether the results could have been biased. The first independent validation was conducted using a multicenter cohort (n = 307) from the international Transbig consortium [13]—none of the patients had received systemic adjuvant therapy. The 70-gene signature was prognostic and identified a low-risk subgroup with 12% distant-metastasis events. Based on these data and other studies [14,15,16], Agendia B.V. developed a prognostic test for commercial use in node-negative breast cancer. The test was later approved by the U.S. Food and Drug Administration. 

In contrast to the Mammaprint assay, the 21-gene recurrence score (RS; Oncotype DX, Genomic Health) was established based on a candidate gene approach in estrogen-receptor positive (ER+) breast cancer patients [17]. The recurrence score is a multiparameter gene expression test that was initially defined in a combined training set of three sample cohorts, including samples from the clinical trial National Surgical Adjuvant Breast and Bowel Project (NSABP)-B20. The finding cohort encompassed a total of 447 node-negative breast cancer patients. In contrast to the Mammaprint assay, the selection of candidate genes was “hypothesis-driven”, and markers were selected due to their known relevance in breast cancer. Sixteen prognostic genes-of-interest were identified, and five reference genes were selected to normalize the gene expression levels. The continuous risk score can be calculated from the relative RNA abundance of the candidate genes. The sources of RNA are formalin-fixed paraffin-embedded (FFPE) tumor blocks. RNA quantification is accomplished by two-step reverse transcription quantitative PCR (RT-qPCR). The 21 gene panel encompasses genes associated with proliferation, invasion, ER and HER2 expression. The proliferation- and HER2-related genes are weighted highest in the mathematical algorithm and, therefore, dominate the test results. The RS can estimate the likelihood of distant metastasis, grouping patients into three risk categories (low, intermediate and high-risk). The RS was validated in the NSABP-B14 trial using 668 node-negative breast cancer patients treated with tamoxifen only [17]. 51% of the evaluated patients from the NSABP-B14 trial were classified as RS-low-risk. This subgroup had a low distant-metastasis rate of 6.8%. Later, the RS was validated in several other clinical trials (NSABP-B20 [18], Southwest Oncology Group (SWOG)-8814 [19], Arimidex, Tamoxifen, Alone or in Combination (ATAC) [20]). The NSABP-B20 results indicated that RS-high-risk patients have a benefit from adjuvant cyclophosphamide, methotrexate and fluorouracil chemotherapy [18]. However, the performance of the RS might be overestimated in this study, since some of the NSABP-B20 samples were included in the training phase of the RS [17,18]. Similar results were reported from the SWOG-8814 study, a randomized trial encompassing node-positive breast cancer patients treated with tamoxifen with or without anthracycline-based chemotherapy treatment [19]. Still, none of the two validation studies were carried out using a non-inferiority design. Accordingly, it remains elusive if the relative benefit in the high-, intermediate- and low-risk groups is really different. Furthermore, both validation studies encompassed HER2-positive patients. In SWOG-8814, it has been shown that the RS is not predictive for chemotherapy benefit in the relevant subgroup of ER+/HER2− patients. For the NSABP-B20 study, no data have been published for this key patient group. Recently, a biomarker substudy of the ATAC trial suggested that centrally-assessed classical clinical parameters, such as ER, progesterone receptor (PgR), HER2 and Ki67, offer the same prognostic information as the recurrence score [21]. 

Both tests—Oncotype DX and Mammaprint—help to determine which patients with early stage breast cancer are at lower risk of recurrence. Both multigene assays are carried out in central reference laboratories in Europe and the USA and have now been used in clinics for several years. Decision impact studies and health economic analyses demonstrated that these first generation signatures can be used to reduce healthcare costs and avoid chemotherapy [22,23,24,25,26,27]. Currently, both tests are prospectively evaluated in the Mindact, RxPonder and TailorX trials, respectively [28,29]. 

## 2. Important Aspects for the Establishment and Clinical Validation of Novel Second Generation Multigene Tests

The substantial increase of knowledge in breast cancer research in the last decade has resulted in a new understanding of how the disease can be managed and how novel drugs and diagnostic tests need to be developed and used in clinical routine. Evaluations of first generation multigene tests for breast cancer did not clearly answer whether or not prognostic tests are fit-for-purpose and should be routinely applied. For instance, the “Evaluation of Genomic Applications in Practice and Prevention (EGAPP) Working Group” found insufficient evidence to make recommendations for or against the use of first generation multigene tests in 2008/2009 [30]. Several research gaps were identified by the EGAPP working group that originated from the study design, analysis and evaluation of the tests. The research gaps were published to encourage further development and evaluation of novel assays [30]. Some of the important aspects for the development of second generation multigene tests are summarized in the following sections. 

### 2.1. Biomarker and Molecular Subtypes

Breast cancer has been recognized to consist of different molecular subtypes. By determining the expression level of ER and HER2, three major subgroups can be defined: ER+/HER2−, ER−/HER2− and HER2+. All three subtypes differ in molecular and clinical characteristics. They are also predictive of patterns of response to systemic treatment or specific targeted agents. For instance, ER+/HER2− breast cancer patients can be treated by antagonizing the activity of estrogen with the selective estrogen-receptor modulator, tamoxifen [31,32]. However, ER+/HER2− breast cancer is a large and heterogeneous subgroup, and frequently, clinical parameters do not allow for deciding whether the patient is sufficiently treated with endocrine therapy only. Combined chemotherapy plus hormonal therapy is, therefore, an additional treatment option. Prognostic tests are urgently needed to allow for tailored treatment strategies in ER+/HER2− breast cancer, since it is well accepted that low absolute risk implies low absolute benefit from the addition of adjuvant chemotherapy [33].

In contrast to that, ER−/HER2− tumors have an increased likelihood of distant recurrence and do not benefit from any targeted intervention developed yet. Chemotherapy is so far the only modality of systemic treatment, and ER−/HER2− tumors seem to benefit the most from cytotoxic regimens [34]. Therefore, almost all patients belonging to this subgroup are currently treated with chemotherapy. Similarly, HER2-overexpressing tumors also show an aggressive behavior, but the clinical outcome can be significantly increased by targeting the extracellular domain of the HER2 receptor using a recombinant monoclonal antibody (trastuzumab) or by HER2 tyrosine kinase inhibitors. ER−/HER2− or HER2+ breast cancer patients were included in the development phase of first generation multigene tests. Therefore, the question was raised whether these assays are of prognostic value once HER2+ and ER−/HER2− breast cancer samples are removed [35]. These first generation multigene tests were never clearly assessed in the different molecular subgroups [36]. Moreover, first generation multigene tests provide little information on ER−/HER2− or HER2+ tumors, since almost all cases in these subgroups are classified as high-risk, due to their general high cell proliferation activity [36]. Biomarkers related to the extracellular environment—especially the adaptive immune system—seem to be more relevant in ER−/HER2− or HER2+ tumors. They appear to be able to identify subgroups of patients with better prognosis or—even more importantly—response to systemic or targeted treatment [37,38,39,40,41]. 

It is unquestionable that molecular subtypes have already begun to alter the way clinical investigators design clinical trials. Specific subgroups of breast cancer patients are enrolled for specific clinical questions. In line with the experiences from the therapeutic trials and daily clinical management, biomarker studies and second generation multigene tests should also be established in a specific molecular subgroup to account for the remarkable differences among groups.

### 2.2. ER+/HER2− Breast Cancer is a Chronic Disease—The Importance of Predicting Late Metastases

The risk of breast cancer recurrence is well known to span more than ten years. However, molecular subtypes differ in terms of timing of distant recurrence. In contrast to ER−/HER2− and HER2+ breast cancer patients, ER+/HER2− breast cancer patients have an increased risk of developing late recurrences for an indefinite period after diagnosis [42,43]. More than 50% of all relapses in ER+/HER2− breast cancer patients occur later than five years after primary treatment. Several large phase III clinical trials have been initiated to study the effects of extended endocrine therapy. Recently, the aTTom (adjuvant Tamoxifen Treatment offer more) and ATLAS (Adjuvant Tamoxifen: Longer Against Shorter) trial reported a significantly improved outcome after completing 10 years of tamoxifen in comparison to five years of tamoxifen treatment [44]. Additionally, the National Cancer Institute of Canada Clinical Trials Group MA-17 [45,46,47,48,49], NSABP-B33 [50] and Austrian Breast and Colorectal Cancer Study Group (ABCSG)-06a [51] trials demonstrated that suppressing estrogen production by an aromatase inhibitor after the discontinuation of tamoxifen therapy prolongs disease-free survival. Currently, there are close to 20,000 additional breast cancer patients treated in randomized phase III clinical trials investigating endocrine therapy of a longer duration. However, the improved outcome observed in the clinical trials needs to be balanced between competing risks/side effects and individual risk of late recurrence. First generation gene expression tests are largely not suitable to predict late metastases [42,52]. Their prognostic performance seems to be time-dependent and higher in the first five years than between five and 10 years of follow-up [52]. Proliferation markers are the principal driving force of first generation tests, and proliferation lacks prognostic value beyond five years of follow-up. Therefore, novel predictors are required to identify patients at very low risk of developing late metastases to safely avoid the side-effects of extended endocrine therapy. 

### 2.3. Pitfalls in Study Design—The Importance of Unique Clinical Characteristics and Treatment Strategies in the Training and Validation Phase

First generation gene signatures were exclusively established in node-negative breast cancer patients. Today, node-positive breast cancer patients with a favorable biology are also strong candidates for omitting chemotherapy treatment. Therefore, several validation studies were initiated to assess whether first generation multigene tests are prognostic in node-positive disease [12,19]. The results of these studies were positive. Still, compared to node-negative breast cancer, the residual risk of recurrence of node-positive patients in the putative low-risk group is considerably higher [19]. This is likely due to the fact that node-positive patients were not included in the training sets of the first generation assays, so the algorithms obtained only exert the technology’s full potential in node-negative patients. 

Another important aspect for the development of multigene assays is that the treatment strategy should be similar in the training and validation cohorts and should be consistent with the current clinical recommendations. Mammaprint, for instance, was developed and validated in an untreated cohort of breast cancer patients [11,12]. However, according to the current clinical guidelines, all ER+ breast cancer patients should be treated with endocrine therapy. Therefore, an easy transfer of the validation results to the current clinical practice may be illusive. 

### 2.4. Additional Prognostic Information—Clinical and Molecular Parameters

Ki-67 is a cellular proliferation marker that has been recently suggested as an immunohistochemistry surrogate to stratify ER+/HER2− breast cancer patients into the intrinsic subgroups, luminal A and luminal B [53]. The St. Gallen consensus panel recommended Ki-67 in 2011 as a marker to decide whether chemotherapy can be safely foregone in patients with ER+/HER2− breast cancer [54]. However, immunohistological determination of Ki-67 expression suffers from intra- and inter-observer variability [55]. The lack of standardization and the unreliable use of a specific cut-off [53] to separate clinical meaningful subgroups has been an obstacle for the marker to make its way from the St. Gallen consensus into major clinical guidelines.

New multigene tests should offer independent prognostic information to all common clinicopathological parameters, including histological markers, like Ki-67. The tests should clearly demonstrate additional prognostic information beyond what can be achieved with standard clinical and histological parameters. The ATAC trial recently suggested that the 21-gene recurrence score offers no additional prognostic information when compared to centrally assessed immunohistochemical parameters, including Ki-67 [21].

Although second generation multigene tests should offer additional prognostic information to all clinicopathological parameters, it seems very unlikely that these tests supplant the significant prognostic information of factors that measure the extent of tumor progression and dissemination. Therefore, molecular information should be refined and complemented with the prognostic information available from clinicopathological parameters [30] to establish hybrid scores integrating classical risk factors offering the best prediction accuracy. 

### 2.5. Decentral Testing—The Importance of Analytic Validity of Tests and External Proficiency Testing

All first generation multigene tests are provided by a diagnostic service through a central manufacturers’ reference laboratory. Due to their high complexity, a standardized robust performance in local routine laboratories seems to be challenging. However, this service model is an obstacle to wide acceptance in Europe’s decentrally organized healthcare systems—not only because of reimbursement issues. 

Therefore, second generation multigene tests should also allow decentralized testing in specialized local laboratories in order to provide a comprehensive tissue-based diagnosis by a pathologist. Therefore, the new tests must be compatible with established clinical workflows. However, for reliable high-quality results, performance characteristics and analytical validation data have to be published, and the robustness of decentralized assays have to be shown in external proficiency testing and round-robin trials.

## 3. The Establishment of EndoPredict—A Second Generation Multigene Test

### 3.1. Relevant Patient Group in Training and Validation

As emphasized before, there is a clinical need for multigene tests to identify those patients with ER+/HER2− breast cancer, who are sufficiently treated with endocrine therapy. Identifying prognostic markers in specific molecular subtypes is pivotal to identifying such patients and has the largest potential to impact clinical decision making. Nevertheless, using the specific group of ER+/HER2− breast cancer patients leads to technical and statistical challenges compared to analyzing pooled patient subgroups.

To this end, training and validation series for the EndoPredict—one of the first second generation multigene tests—were carefully selected. A large high-throughput microarray-based screening study was conducted to establish the EndoPredict signature [56]. For training, ER+/HER2− breast cancer samples were selected from four different institutes and two large clinical trials. ER−/HER2− or HER2+ breast cancer patients were excluded from the training and validation series. Additionally, tumor samples were collected from patients with and without axillary lymph-node involvement. All patients were uniquely treated with endocrine therapy only. 

### 3.2. Gene Selection and Algorithm Design

Affymetrix HG-U133A microarrays were employed to identify the most relevant prognostic marker genes. The microarray platform is a highly valuable and reliable tool to discover differentially expressed genes. Initial concerns regarding the reproducibility of microarray experiments and mathematical approaches to select candidates had been addressed by the Microarray Quality Control (MAQC) consortium. The MAQC consortium clearly showed that microarrays are useful for identifying differentially expressed markers [57,58,59]. 

**Figure 1:**
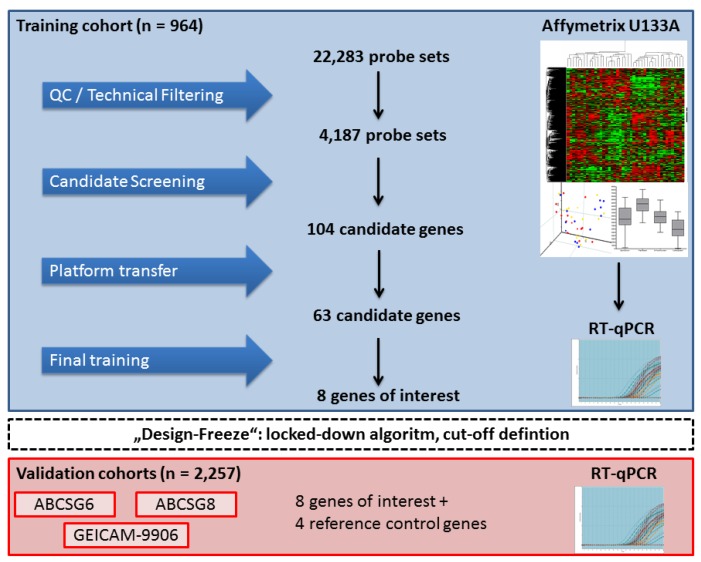
Microarray-based screening and platform transfer in the training phase. QC, Quality Control; RT-qPCR, reverse transcription quantitative PCR.

There are different approaches for how to discover a clinically relevant gene signature using high-dimensional gene expression data [60]. The “top-down” approach was used to establish the 70-gene signature (Mammaprint). This is a purely statistical approach by simply looking for genes that are associated with clinical outcome independent of any biological or clinical assumptions. In contrast to that, the “bottom-up” strategy can be employed. It is based on establishing a gene expression signature according to a hypothesis, a specific biological subgroup or a clinical phenotype [60]. Subsequently, the signature is tested against clinical outcome information. Sotiriou and colleagues used the “bottom-up” strategy to establish the Genomic Grade Index (GGI). GGI was established to distinguish the large subgroup of intermediate-grade (grade 2) tumors [61] into prognostic subgroups by using large-scale gene expression profiling data [62]. The GGI signature was prognostic in independent data sets and able to refine the histological grade assessment [63,64]. A “top-down” approach was used at the beginning of the EndoPredict development to screen for prognostic markers. Sequential screening steps were used, and marker lists were continuously reduced to construct a robust final algorithm (Figure 1) [56]. First, gene expression levels assessed by different probe sets were quality controlled, and informative genes were selected using stringent technical filter criteria. Genes with a low expression level or a low dynamic range were omitted from any further analysis. Afterwards, gene candidates were selected that were consistently associated with prognosis using Cox regression in ER+/HER2− breast cancer patients. The first marker set was further enriched by adding candidate genes that are known to be of particular relevance in breast cancer. Additionally, marker genes were analyzed by unsupervised clustering and principal component analysis to elucidate the association of single markers, gene modules and clinical characteristics. Marker genes were also used for bivariate Cox regression analysis using the gene expressions levels of the proliferation marker, TOP2A. The results of the bivariate analysis could help to identify prognostic genes that are not associated with cell cycle processes. Finally, marker genes were selected according to multiple parameters: prognostic performance in univariate and bivariate Cox regression, analytical performance and associated gene modules. In a nutshell, the microarray-based screening study to define EndoPredict was a combination of a top-down approach and a hypothesis-driven candidate selection. 

Although microarray-based gene expression analysis has evolved dramatically, the technology has not found its way into clinical routine. This is particularly due to the fact that microarrays work best on RNA from fresh-frozen tumor samples. However, the collection and storage of fresh-frozen samples is associated with logistical challenges and may not be applicable outside large and optimally equipped clinical centers. In contrast to that, FFPE tissue sections are generally prepared from every single tumor for its histopathological assessment. There are emerging technologies to carry out gene expression profiling using FFPE tissue and microarrays, but data quality is still an issue, due to the short RNA fragments created by tissue fixation. RT-qPCR is an appreciated alternative to reliably measure candidate genes using FFPE tissue sections. Robust protocols have been established to automatically extract RNA from FFPE tissue sections [65,66,67]. 

In order to avoid workflow issues associated with fresh tissue, 104 candidate genes from the EndoPredict microarray-based screening studies were transferred from fresh-frozen tissue and microarrays to FFPE and RT-qPCR. This was an essential step to move the promising candidates from the research laboratory stage to clinical validation and application. The final EndoPredict score was established using 63 marker genes that showed a considerably high correlation between microarrays and the RT-qPCR platform. Eight genes of interest were selected for the EndoPredict score. Besides proliferation, the genes chosen cover several cellular processes, such as apoptosis, DNA repair, cell adhesion and cell signaling. Nevertheless, the markers are also co-regulated with genes reflecting two relevant biological modules known to contribute to recurrence risk: proliferation and ER− signaling/differentiation [56,68].

Since breast cancer is a complex disease; even the best gene expression profile cannot mirror the whole clinical course of the disease. Nodal status and tumor size are still important clinical variables that are independently associated with prognosis. Therefore, improvement of prediction accuracy is possible using a multidimensional approach able to integrate tumor biology and disease burden. This had already been a requirement posted by the EGAPP group [30]. The molecular information of EndoPredict was consequently combined with the clinical parameter nodal status and tumor size, resulting in the molecular and clinical risk score, EndoPredict-clin (EPclin). 

### 3.3. Independent Clinical Validation of the EndoPredict Test

Complex high-dimensional gene expression data sets are prone to overfitting, since many more explanatory variables per tumor samples are commonly collected than the number of samples used to generate the dataset. As a consequence, mathematical algorithms perfectly suitable in the training set may subsequently fail in another test set [69]. Therefore, multigene tests should be tested and confirmed using independent validation studies only employing samples not used in the definition phase of the mathematical algorithm. 

**Figure 2:**
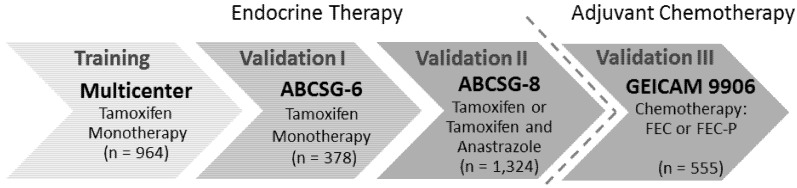
Training and independent validation series for the EndoPredict test. FEC, fluorouracil, epirubicin and cyclophosphamide; FEC-P, fluorouracil, epirubicin and cyclophosphamide followed by weekly paclitaxel.

To this end, the predefined and locked-down EndoPredict test—including all cut-off values—was validated in three independent clinical trials (n = 2,257, Figure 2). First, EndoPredict was assessed in postmenopausal ER+/HER2− breast cancer patients from the ABCG-6 trial, immediately followed by the same analysis in the ABCSG-8 trial. All patients had been treated with tamoxifen or tamoxifen, followed by anastrozole (ABCSG-6 [n = 378]; ABCSG-8 [n = 1,324]) [56,70]. None of the patients had been treated with adjuvant chemotherapy. EndoPredict was analyzed retrospectively in both phase III trials relying on prospectively pre-specified study objectives and laboratory data, as recommended by Simon *et al.* [71]. This allows one to generate level I evidence using this “prospective-retrospective” approach, with consistent results in at least two validation studies. Both validation series were blinded to any clinical outcome until the mathematical model and cut-offs had been locked down and the test had been applied to all samples. A statistical analysis plan had been specified before the performance of the assay was evaluated. 

These trials demonstrated that EndoPredict can precisely identify distant metastases in patient cohorts treated with endocrine therapy only. The EPclin low-risk group had an excellent prognosis, with an estimated risk of recurrence below 5% in both studies [56]. Treatment recommendations are commonly based upon risk of recurrence and the estimated benefits of treatment weighted against adverse events of therapy. Due to the low-risk of recurrence, the absolute benefit of adjuvant chemotherapy does not outweigh its medical risks and adverse events affecting quality of life. Therefore, a risk of 5% allows one to safely forgo chemotherapy [29]. Additionally, the study demonstrated that EndoPredict adds prognostic performance beyond all common clinicopathological parameters, including centrally-analyzed Ki-67 and quantitative ER. 

A sub-analysis of the ABCSG trials demonstrated that EndoPredict identifies early and late distant metastases [68]. The EP score provided additional prognostic information regarding late recurrence beyond what can be achieved by all common clinical parameters. An explorative analysis of the biological modules enclosed in the EndoPredict score demonstrated that proliferation-associated genes add prognostic information regarding early relapses, but show a less prognostic performance to identify late recurrence events. In contrast to that, ER-signaling genes add additional prognostic information to all clinical parameters for predicting late metastases. Additionally, the EPclin score improved the prognostic performance for predicting late recurrences: EPclin-low-risk patients had an absolute risk of distant metastasis of 1.8% during the period of five years after the end of endocrine therapy. This provides EndoPredict with the potential to identify patients expected to gain little benefit from extended endocrine treatment. The competing health risk of the individual patient needs to be balanced against the observed risk estimation of EndoPredict to decide on extended treatment strategies.

The EndoPredict test was also clinically validated in node-positive ER+/HER2− breast cancer patients treated with chemotherapy [72]. This was the third independent “prospective-retrospective” validation in a large biomarker cohort. So far, only a few of the available prognostic tests have been validated in studies enrolling node-positive patients only [19]. Most of these studies showed that the tests also allow one to identify subgroups with a fair prognosis in spite of nodal involvement. However, the putative low-risk patients still have a considerably risk of disease. It may exceed 30% likelihood of distant metastasis within 10 years [19]. In contrast to that, the EndoPredict validation in the Grupo Español de Investigación en Cáncer de Mama (GEICAM)-9906 trial demonstrated that EndoPredict-low-risk patients had a 10-year risk of recurrence below 10%. Multivariate analysis showed that EndoPredict provides additional prognostic information to common clinical variables. The results suggest that EndoPredict provides important information regarding the residual risk of recurrence after a modern, anthracycline and taxane-based regimen of chemotherapy. While the initial validation studies for EndoPredict (ABCSG6 and ABCSG8) only encompassed postmenopausal breast cancer patients, the analysis of the GEICAM-9906 study clearly demonstrated that EndoPredict is prognostic in pre- and post-menopausal breast cancer patients. The excellent prognostic performance in premenopausal patients suggests that EndoPredict can be also used for risk stratification of younger women. Nevertheless, these results should be taken with some care, since all patients in the GEICAM-9906 trial were treated with chemotherapy. 

EndoPredict was also tested in a neo-adjuvant cohort of ER+/HER2− breast cancer patients treated with anthracycline/taxane-based therapy [73]. Almost all pathological complete response (pCR) events (91%) were classified as EP-high-risk, suggesting that EP-low-risk tumors are resistant towards chemotherapy treatment and do not particularly benefit from cytotoxic therapy. EndoPredict is currently prospectively tested in a neo-adjuvant trial (ABCSG-34) to validate these results. Neo-adjuvant studies are well suited to analyze chemotherapy response in different subgroups, and FFPE tissue, as well as response data is often readily available. Assessing chemotherapy in adjuvant trials is more demanding, as it requires a study with two treatment arms comparing an endocrine and endo-chemotherapy regimen. For ER+/HER2− breast cancer, this type of study has never been properly completed, yet, and published for any RNA-based multigene assay. Available data for ER+/HER2− patients is either insignificant [19] or tainted with results from more aggressive, non-luminal tumors [18].

### 3.4. Analytical Validation and Proficiency Testing

Before diagnostic tests are ready for wide-scale clinical application, an extensive analytical validation is necessary to ensure analytical validity and high technical reproducibility within and between laboratories. The EndoPredict test was developed according to current clinical laboratory standards. A comprehensive analytical validation study was carried out to demonstrate that EndoPredict allows a robust and precise determination of gene expression levels [74]. The analytical validation was conducted in accordance to the recommendations of the Clinical Laboratory Standards Institute (CLSI). Different breast cancer samples were used in this study to evaluate essential analytical parameters, like RNA input range, limit of detection, precision and inter-laboratory variability. Finally, the analytical parameters were verified in a molecular pathology laboratory, and the results clearly showed that there was no difference in test performance when compared to the manufacturer’s claims. A proficiency testing program with seven molecular pathology laboratories was subsequently initiated to finally prove that EndoPredict showed reproducible performance with good precision and negligible laboratory-to-laboratory variations [75]. The study demonstrated that EndoPredict is the first multigene test for breast cancer patients that can be reliably used in the decentralized setting [75]. EndoPredict seems to be more reproducible than immunohistochemical tests that reported variations in the decentralized setting, due to intra- and inter-laboratory disconcordance [55,76,77]. 

EndoPredict results were also compared between core biopsies and surgical tissue specimens in a further analytical study [78]. Test results were highly correlated between core biopsies and surgical specimens, indicating that the assay can also be used on core biopsy samples. The study also showed that inflammatory changes induced by biopsy sampling do not affect the test result [78]. This is probably due to the fact that EndoPredict does not contain genes directly associated with inflammation or wound healing. Therefore, tumor areas containing preoperative biopsy-induced changes might be also used to determine the EndoPredict score, obliterating the need for any biopsy channel dissection that may be associated with less robust assays.

### 3.5. Clinical Utility—Comparison to Clinical Guidelines, Decision Impact and Health Economics

The stratification power of three widely accepted international guidelines (German S3 [79], National Comprehensive Cancer Network (NCCN) [80], St. Gallen [54]) were compared with the EndoPredict in 1,702 ER+/HER2− breast cancer patients treated with endocrine therapy alone [81]. All guidelines and EndoPredict identified a low-risk subgroup with excellent prognosis and a metastasis rate of approximately 5% after 10 years of follow-up. However, the three guidelines only assigned 7–19% of the patients to a low-risk group. In contrast, EndoPredict stratified 63% of the analyzed cohort as low-risk. This is an indication of EndoPredict’s higher specificity. Especially patients classified as intermediate/high-risk by clinical guidelines were reclassified by EndoPredict. The results clearly showed that EndoPredict outperformed all conventional parameters and guidelines by identifying a larger set of low-risk patient’s not needing cytotoxic treatment. Overall, EndoPredict seems to identify those women who should or should not receive chemotherapy and could ensure that more women receive the appropriate treatment.

The Charité University Hospital recently analyzed the performance of the EndoPredict test and performed a prospective assessment of the impact on treatment decisions in 167 breast cancer cases [82]. The comparison of the treatment decisions before and after knowledge of the EndoPredict test result indicated that 37.7% of all evaluated breast cancer patients received a different adjuvant treatment recommendation as originally made, on the basis of clinical factors alone. 12% of patients were routed to an additional chemotherapy, thus avoiding potential under-treatment, while 25% of patients were directed to endocrine therapy alone, thus avoiding overtreatment. The results were supported by an evaluation conducted at the interdisciplinary breast center of the Technical University of Munich [83]. The decision impact study was carried out to prospectively examine whether EndoPredict affects the oncologist’s and patient’s adjuvant treatment choice. The results of this study also indicated that EndoPredict can indeed change treatment selection beyond standard clinical parameters and adds value to decision making in comparison to guideline-based patient management. Using the EndoPredict test results, in 44% of the 123 consecutive cases of ER+/HER2−, the breast cancer patients’ adjuvant chemotherapy was omitted. The results of both decision impact studies show that chemotherapy treatment can be markedly reduced with EndoPredict. Accordingly, unnecessary side effects and their corresponding costs can be reduced, as well. 

Indeed, a health economics analysis further proved that the combination of clinical guidelines and EndoPredict significantly reduced the costs associated with managing primary breast cancer and leads to improved “quality adjusted life years” (QALY) [84]. Overall, the use of EndoPredict led to a reduction of treatment costs. 

## 4. EndoPredict—Ready for Prime Time?

**Figure 3:**
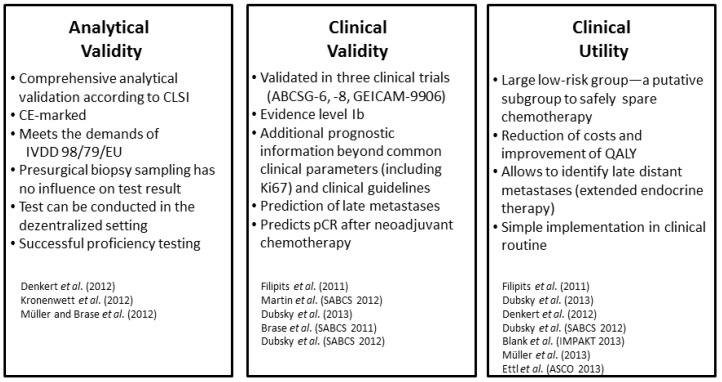
Summary of the important aspects regarding analytical and clinical validity, as well as clinical utility. CLSI, Clinical Laboratory Standards Institute; CE, Communauté européenne; IVDD, *In vitro* Diagnostic Directive; EU, European Union; pCR, pathological complete response; QALY, quality adjusted life years.

As mentioned earlier, the “Evaluation of Genomic Applications in Practice and Prevention (EGAPP) Working Group” evaluated first generation multigene tests in 2009. The EGAPP working group concluded that first generation multigene tests should not be regularly applied in clinical routine, since the benefit and risk cannot be reliably assessed [30]. The foregoing sections showed that EndoPredict addressed several research gaps with regard to clinical validity, analytical validity and clinical utility. The EndoPredict test has been validated in three independent prospective-retrospective clinical trials. All studies were carried out in the prospective-retrospective design (category B studies), resulting in a level of evidence of I, according to Simon *et al.* [71]. This evidence level has been also acknowledged in German guidelines, as well as by international experts [85]. EndoPredict integrates classical risk factors, such as tumor size and nodal status, into a molecular-clinicopathological hybrid score and predicts not only early, but also late, metastasis. EndoPredict is so far the first multigene test that has been successfully validated in proficiency testing, allowing a widespread adoption in routine laboratory work-up of the molecular pathology. Toxicities of chemotherapy treatment can be safely avoided, and costs and quality-of-life issues can be considerably changed. Figure 3 summarizes all important aspects that demonstrated the high clinical and analytical validity, as well as the clinical utility of this multigene test. 

Therefore, a novel evaluation of all first and second generation multigene tests—including EndoPredict—by the EGAPP working group would be desirable in the near future. 

## 5. Perspectives

This review demonstrates that a carefully designed and executed series of training, validation and analytical studies is required to transfer a microarray-based gene signature to a clinically useful test. The rapid gain of knowledge in breast cancer diagnosis and therapy has identified additional clinical needs that the development process of new multigene tests has to account for. Here, we focused on the EndoPredict test, but there are also other novel second generation multigene tests that have been recently established. PAM50 and the breast cancer index (BCI), for instance, can be also used to identify patients with low risk of recurrence [86,87,88,89]. Both tests can be also applied for predicting late recurrence events [52]. Although prognostic multigene tests comprise different gene sets, all mentioned gene signatures seem to have prognostic value and single out similar subsets of breast cancer patients. Nevertheless, there is still discordance in risk stratification [90], and multigene tests should be directly compared using the same clinical material to allow an estimation of the performance characteristics. Data from the ATAC trial suggested that PAM50 offers more prognostic information than the 21-gene recurrence score [91].

So far, first and second generation multigene assays help to determine which patients with early stage breast cancer are at lower risk of recurrence, allowing women to safely forgo chemotherapy treatment. In contrast to that, they have no ability to predict the most appropriate treatment scenario in high-risk patients. All multigene tests investigated so far have failed to identify a subgroup with a particular benefit from adding paclitaxel to anthracycline-based chemotherapy treatment [72,92]. Predictive markers for specific cytotoxic agents are needed to select the tailored treatment strategies for high-risk breast cancer patients. Currently, none of the identified predictive markers for selecting individualized chemotherapy strategies in breast cancer has been successfully validated. Additionally, companion diagnostic tests could help to identify subsets of patients likely to respond to novel targeted treatment strategies. 

The review article has summarized several important steps to be considered to successfully establish, validate and use second generation multigene tests. The process should be generally applicable to transferring other microarray signatures from the research laboratory to clinical practice.

## References

[1] Siegel R., Naishadham D., Jemal A. (2013). Cancer statistics, 2013. CA. Cancer J. Clin..

[2] Olivotto I.A., Bajdik C.D., Ravdin P.M., Speers C.H., Coldman A.J., Norris B.D., Davis G.J., Chia S.K., Gelmon K.A. (2005). Population-based validation of the prognostic model ADJUVANT! for early breast cancer. J. Clin. Oncol..

[3] Galea M.H., Blamey R.W., Elston C.E., Ellis I.O. (1992). The Nottingham Prognostic Index in primary breast cancer. Breast Cancer Res. Treat..

[4] D’Eredita G., Giardina C., Martellotta M., Natale T., Ferrarese F. (2001). Prognostic factors in breast cancer: The predictive value of the Nottingham Prognostic Index in patients with a long-term follow-up that were treated in a single institution. Eur. J. Cancer.

[5] Goldhirsch A., Ingle J.N., Gelber R.D., Coates A.S., Thurlimann B., Senn H.J. (2009). Thresholds for therapies: Highlights of the St. Gallen International Expert Consensus on the primary therapy of early breast cancer 2009. Ann. Oncol..

[6] Perou C.M., Sorlie T., Eisen M.B., van de Rijn M., Jeffrey S.S., Rees C.A., Pollack J.R., Ross D.T., Johnsen H., Akslen L.A. (2000). Molecular portraits of human breast tumours. Nature.

[7] Sorlie T., Perou C.M., Tibshirani R., Aas T., Geisler S., Johnsen H., Hastie T., Eisen M.B., van de Rijn M., Jeffrey S.S. (2001). Gene expression patterns of breast carcinomas distinguish tumor subclasses with clinical implications. Proc. Natl. Acad. Sci. USA.

[8] Sorlie T., Tibshirani R., Parker J., Hastie T., Marron J.S., Nobel A., Deng S., Johnsen H., Pesich R., Geisler S. (2003). Repeated observation of breast tumor subtypes in independent gene expression data sets. Proc. Natl. Acad. Sci. USA.

[9] Rouzier R., Perou C.M., Symmans W.F., Ibrahim N., Cristofanilli M., Anderson K., Hess K.R., Stec J., Ayers M., Wagner P. (2005). Breast cancer molecular subtypes respond differently to preoperative chemotherapy. Clin. Cancer Res..

[10] Gluck S., de Snoo F., Peeters J., Stork-Sloots L., Somlo G. (2013). Molecular subtyping of early-stage breast cancer identifies a group of patients who do not benefit from neoadjuvant chemotherapy. Breast Cancer Res. Treat..

[11] Van’t Veer L.J., Dai H., van de Vijver M.J., He Y.D., Hart A.A., Mao M., Peterse H.L., van der Kooy K., Marton M.J., Witteveen A.T. (2002). Gene expression profiling predicts clinical outcome of breast cancer. Nature.

[12] Van de Vijver M.J., He Y.D., van’t Veer L.J., Dai H., Hart A.A., Voskuil D.W., Schreiber G.J., Peterse J.L., Roberts C., Marton M.J. (2002). A gene-expression signature as a predictor of survival in breast cancer. N. Engl. J. Med..

[13] Buyse M., Loi S., van’t Veer L., Viale G., Delorenzi M., Glas A.M., d’Assignies M.S., Bergh J., Lidereau R., Ellis P. (2006). Validation and clinical utility of a 70-gene prognostic signature for women with node-negative breast cancer. J. Natl. Cancer Inst..

[14] Mook S., Schmidt M.K., Weigelt B., Kreike B., Eekhout I., van de Vijver M.J., Glas A.M., Floore A., Rutgers E.J., van’t Veer L.J. (2010). The 70-gene prognosis signature predicts early metastasis in breast cancer patients between 55 and 70 years of age. Ann. Oncol..

[15] Wittner B.S., Sgroi D.C., Ryan P.D., Bruinsma T.J., Glas A.M., Male A., Dahiya S., Habin K., Bernards R., Haber D.A. (2008). Analysis of the MammaPrint breast cancer assay in a predominantly postmenopausal cohort. Clin. Cancer Res..

[16] Glas A.M., Floore A., Delahaye L.J., Witteveen A.T., Pover R.C., Bakx N., Lahti-Domenici J.S., Bruinsma T.J., Warmoes M.O., Bernards R. (2006). Converting a breast cancer microarray signature into a high-throughput diagnostic test. BMC Genomics.

[17] Paik S., Shak S., Tang G., Kim C., Baker J., Cronin M., Baehner F.L., Walker M.G., Watson D., Park T. (2004). A multigene assay to predict recurrence of tamoxifen-treated, node-negative breast cancer. N. Engl. J. Med..

[18] Paik S., Tang G., Shak S., Kim C., Baker J., Kim W., Cronin M., Baehner F.L., Watson D., Bryant J. (2006). Gene expression and benefit of chemotherapy in women with node-negative, estrogen receptor-positive breast cancer. J. Clin. Oncol..

[19] Albain K.S., Barlow W.E., Shak S., Hortobagyi G.N., Livingston R.B., Yeh I.T., Ravdin P., Bugarini R., Baehner F.L., Davidson N.E. (2010). Prognostic and predictive value of the 21-gene recurrence score assay in postmenopausal women with node-positive, oestrogen-receptor-positive breast cancer on chemotherapy: A retrospective analysis of a randomised trial. Lancet Oncol..

[20] Dowsett M., Cuzick J., Wale C., Forbes J., Mallon E.A., Salter J., Quinn E., Dunbier A., Baum M., Buzdar A. (2010). Prediction of risk of distant recurrence using the 21-gene recurrence score in node-negative and node-positive postmenopausal patients with breast cancer treated with anastrozole or tamoxifen: A TransATAC study. J. Clin. Oncol..

[21] Cuzick J., Dowsett M., Pineda S., Wale C., Salter J., Quinn E., Zabaglo L., Mallon E., Green A.R., Ellis I.O. (2011). Prognostic value of a combined estrogen receptor, progesterone receptor, Ki-67, and human epidermal growth factor receptor 2 immunohistochemical score and comparison with the Genomic Health recurrence score in early breast cancer. J. Clin. Oncol..

[22] Klang S.H., Hammerman A., Liebermann N., Efrat N., Doberne J., Hornberger J. (2010). Economic implications of 21-gene breast cancer risk assay from the perspective of an Israeli-managed health-care organization. Value Health.

[23] Partin J.F., Mamounas E.P. (2011). Impact of the 21-gene recurrence score assay compared with standard clinicopathologic guidelines in adjuvant therapy selection for node-negative, estrogen receptor-positive breast cancer. Ann. Surg. Oncol..

[24] Bueno-de-Mesquita J.M., van Harten W.H., Retel V.P., van’t Veer L.J., van Dam F.S., Karsenberg K., Douma K.F., van Tinteren H., Peterse J.L., Wesseling J. (2007). Use of 70-gene signature to predict prognosis of patients with node-negative breast cancer: A prospective community-based feasibility study (RASTER). Lancet Oncol..

[25] Geffen D.B., Amir N., Sion-Vardy N., Ariad S., Kachko L., Bayme M., Delgado B., Dyomin V., Argov S., Koretz M. (2009). Stage I breast cancer in a regional oncology practice in Israel 2002–2006: Clinicopathologic features, risk estimation and planned therapy of 328 consecutive patients. Breast.

[26] Asad J., Jacobson A.F., Estabrook A., Smith S.R., Boolbol S.K., Feldman S.M., Osborne M.P., Boachie-Adjei K., Twardzik W., Tartter P.I. (2008). Does oncotype DX recurrence score affect the management of patients with early-stage breast cancer?. Am. J. Surg..

[27] Drukker C.A., Bueno-de-Mesquita J.M., Retel V.P., van Harten W.H., van Tinteren H., Wesseling J., Roumen R.M., Knauer M., van’t Veer L.J., Sonke G.S. (2013). A prospective evaluation of a breast cancer prognosis signature in the observational RASTER study. Int. J. Cancer.

[28] Bogaerts J., Cardoso F., Buyse M., Braga S., Loi S., Harrison J.A., Bines J., Mook S., Decker N., Ravdin P. (2006). Gene signature evaluation as a prognostic tool: Challenges in the design of the MINDACT trial. Nat. Clin. Pract. Oncol..

[29] Cardoso F., van’t Veer L., Rutgers E., Loi S., Mook S., Piccart-Gebhart M.J. (2008). Clinical application of the 70-gene profile: the MINDACT trial. J. Clin. Oncol..

[30] Evaluation of Genomic Applications in Practice and Prevention (EGAPP) Working Group (2009). Recommendations from the EGAPP working group: Can tumor gene expression profiling improve outcomes in patients with breast cancer?. Genet. Med..

[31] Fisher B., Dignam J., Bryant J., Wolmark N. (2001). Five *versus* more than five years of tamoxifen for lymph node-negative breast cancer: Updated findings from the National Surgical Adjuvant Breast and Bowel Project B-14 randomized trial. J. Natl. Cancer Inst..

[32] Fisher B., Jeong J.H., Bryant J., Anderson S., Dignam J., Fisher E.R., Wolmark N. (2004). Treatment of lymph-node-negative, oestrogen-receptor-positive breast cancer: Long-term findings from National Surgical Adjuvant Breast and Bowel Project randomised clinical trials. Lancet.

[33] Peto R., Davies C., Godwin J., Gray R., Pan H.C., Clarke M., Cutter D., Darby S., McGale P., Taylor C. (2012). Comparisons between different polychemotherapy regimens for early breast cancer: Meta-analyses of long-term outcome among 100,000 women in 123 randomised trials. Lancet.

[34] Berry D.A., Cirrincione C., Henderson I.C., Citron M.L., Budman D.R., Goldstein L.J., Martino S., Perez E.A., Muss H.B., Norton L. (2006). Estrogen-receptor status and outcomes of modern chemotherapy for patients with node-positive breast cancer. JAMA.

[35] Milburn M., Rosman M., Mylander C., Tafra L. (2013). Is oncotype DX recurrence score (RS) of prognostic value once HER2-positive and low-ER expression patients are removed?. Breast J..

[36] Desmedt C., Haibe-Kains B., Wirapati P., Buyse M., Larsimont D., Bontempi G., Delorenzi M., Piccart M., Sotiriou C. (2008). Biological processes associated with breast cancer clinical outcome depend on the molecular subtypes. Clin. Cancer Res..

[37] Schmidt M., Bohm D., von Torne C., Steiner E., Puhl A., Pilch H., Lehr H.A., Hengstler J.G., Kolbl H., Gehrmann M. (2008). The humoral immune system has a key prognostic impact in node-negative breast cancer. Cancer Res..

[38] Schmidt M., Hellwig B., Hammad S., Othman A., Lohr M., Chen Z., Boehm D., Gebhard S., Petry I., Lebrecht A. (2012). A comprehensive analysis of human gene expression profiles identifies stromal immunoglobulin Ðº C as a compatible prognostic marker in human solid tumors. Clin. Cancer Res..

[39] Schmidt M., Hengstler J.G., von Torne C., Koelbl H., Gehrmann M.C. (2009). Coordinates in the universe of node-negative breast cancer revisited. Cancer Res..

[40] Teschendorff A.E., Gomez S., Arenas A., El-Ashry D., Schmidt M., Gehrmann M., Caldas C. (2010). Improved prognostic classification of breast cancer defined by antagonistic activation patterns of immune response pathway modules. BMC Cancer.

[41] Bianchini G., Qi Y., Alvarez R.H., Iwamoto T., Coutant C., Ibrahim N.K., Valero V., Cristofanilli M., Green M.C., Radvanyi L. (2010). Molecular anatomy of breast cancer stroma and its prognostic value in estrogen receptor-positive and -negative cancers. J. Clin. Oncol..

[42] Esserman L.J., Moore D.H., Tsing P.J., Chu P.W., Yau C., Ozanne E., Chung R.E., Tandon V.J., Park J.W., Baehner F.L. (2011). Biologic markers determine both the risk and the timing of recurrence in breast cancer. Breast Cancer Res. Treat..

[43] Jatoi I., Anderson W.F., Jeong J.H., Redmond C.K. (2011). Breast cancer adjuvant therapy: Time to consider its time-dependent effects. J. Clin. Oncol..

[44] Davies C., Pan H., Godwin J., Gray R., Arriagada R., Raina V., Abraham M., Alencar V.H., Badran A., Bonfill X. (2013). Long-term effects of continuing adjuvant tamoxifen to 10 years *versus* stopping at 5 years after diagnosis of oestrogen receptor-positive breast cancer: ATLAS, a randomised trial. Lancet.

[45] Goss P.E. (2007). Letrozole in the extended adjuvant setting: MA.17. Breast Cancer Res. Treat..

[46] Goss P.E., Ingle J.N., Martino S., Robert N.J., Muss H.B., Livingston R.B., Davidson N.E., Perez E.A., Chavarri-Guerra Y., Cameron D.A. (2013). Impact of premenopausal status at breast cancer diagnosis in women entered on the placebo-controlled NCIC CTG MA17 trial of extended adjuvant letrozole. Ann. Oncol..

[47] Goss P.E., Ingle J.N., Martino S., Robert N.J., Muss H.B., Piccart M.J., Castiglione M., Tu D., Shepherd L.E., Pritchard K.I. (2005). Randomized trial of letrozole following tamoxifen as extended adjuvant therapy in receptor-positive breast cancer: Updated findings from NCIC CTG MA.17. J. Natl. Cancer Inst..

[48] Goss P.E., Ingle J.N., Martino S., Robert N.J., Muss H.B., Piccart M.J., Castiglione M., Tu D., Shepherd L.E., Pritchard K.I. (2007). Efficacy of letrozole extended adjuvant therapy according to estrogen receptor and progesterone receptor status of the primary tumor: National Cancer Institute of Canada Clinical Trials Group MA.17. J. Clin. Oncol..

[49] Goss P.E., Ingle J.N., Martino S., Robert N.J., Muss H.B., Piccart M.J., Castiglione M., Tu D., Shepherd L.E., Pritchard K.I. (2003). A randomized trial of letrozole in postmenopausal women after five years of tamoxifen therapy for early-stage breast cancer. N. Engl. J. Med..

[50] Mamounas E.P., Jeong J.H., Wickerham D.L., Smith R.E., Ganz P.A., Land S.R., Eisen A., Fehrenbacher L., Farrar W.B., Atkins J.N. (2008). Benefit from exemestane as extended adjuvant therapy after 5 years of adjuvant tamoxifen: Intention-to-treat analysis of the National Surgical Adjuvant Breast And Bowel Project B-33 trial. J. Clin. Oncol..

[51] Jakesz R., Greil R., Gnant M., Schmid M., Kwasny W., Kubista E., Mlineritsch B., Tausch C., Stierer M., Hofbauer F. (2007). Extended adjuvant therapy with anastrozole among postmenopausal breast cancer patients: Results from the randomized Austrian Breast and Colorectal Cancer Study Group Trial 6a. J. Natl. Cancer Inst..

[52] Sgroi D.C., Sestak I., Cuzick J., Zhang Y., Schnabel C.A., Erlander M.G., Goss P.E., Dowsett M. (2012). Comparative performance of breast cancer Index (BCI) *vs*. oncotype Dx and IHC4 in the prediction of late recurrence in hormonal receptor-positive lymph node-negative breast cancer patients: A TransATAC study. Cancer Res..

[53] Cheang M.C., Chia S.K., Voduc D., Gao D., Leung S., Snider J., Watson M., Davies S., Bernard P.S., Parker J.S. (2009). Ki67 index, HER2 status, and prognosis of patients with luminal B breast cancer. J. Natl. Cancer Inst..

[54] Goldhirsch A., Wood W.C., Coates A.S., Gelber R.D., Thurlimann B., Senn H.J. (2011). Strategies for subtypes—Dealing with the diversity of breast cancer: Highlights of the St. Gallen International Expert Consensus on the Primary Therapy of Early Breast Cancer 2011. Ann. Oncol..

[55] Varga Z., Diebold J., Dommann-Scherrer C., Frick H., Kaup D., Noske A., Obermann E., Ohlschlegel C., Padberg B., Rakozy C. (2012). How reliable is Ki-67 immunohistochemistry in grade 2 breast carcinomas? A QA study of the Swiss Working Group of Breast- and Gynecopathologists. PLoS One.

[56] Filipits M., Rudas M., Jakesz R., Dubsky P., Fitzal F., Singer C.F., Dietze O., Greil R., Jelen A., Sevelda P. (2011). A new molecular predictor of distant recurrence in ER-positive, HER2-negative breast cancer adds independent information to conventional clinical risk factors. Clin. Cancer Res..

[57] Patterson T.A., Lobenhofer E.K., Fulmer-Smentek S.B., Collins P.J., Chu T.M., Bao W., Fang H., Kawasaki E.S., Hager J., Tikhonova I.R. (2006). Performance comparison of one-color and two-color platforms within the MicroArray Quality Control (MAQC) project. Nat. Biotechnol..

[58] Shi L., Reid L.H., Jones W.D., Shippy R., Warrington J.A., Baker S.C., Collins P.J., de Longueville F., Kawasaki E.S., Lee K.Y. (2006). The MicroArray Quality Control (MAQC) project shows inter- and intraplatform reproducibility of gene expression measurements. Nat. Biotechnol..

[59] Irizarry R.A., Warren D., Spencer F., Kim I.F., Biswal S., Frank B.C., Gabrielson E., Garcia J.G., Geoghegan J., Germino G. (2005). Multiple-laboratory comparison of microarray platforms. Nat. Methods.

[60] Sotiriou C., Pusztai L. (2009). Gene-expression signatures in breast cancer. N. Engl. J. Med..

[61] Furness P.N., Taub N., Assmann K.J., Banfi G., Cosyns J.P., Dorman A.M., Hill C.M., Kapper S.K., Waldherr R., Laurinavicius A. (2003). International variation in histologic grading is large, and persistent feedback does not improve reproducibility. Am. J. Surg. Pathol..

[62] Sotiriou C., Wirapati P., Loi S., Harris A., Fox S., Smeds J., Nordgren H., Farmer P., Praz V., Haibe-Kains B. (2006). Gene expression profiling in breast cancer: Understanding the molecular basis of histologic grade to improve prognosis. J. Natl. Cancer Inst..

[63] Desmedt C., Giobbie-Hurder A., Neven P., Paridaens R., Christiaens M.R., Smeets A., Lallemand F., Haibe-Kains B., Viale G., Gelber R.D. (2009). The Gene expression Grade Index: A potential predictor of relapse for endocrine-treated breast cancer patients in the BIG 1–98 trial. BMC Med. Genom..

[64] Loi S., Haibe-Kains B., Desmedt C., Lallemand F., Tutt A.M., Gillet C., Ellis P., Harris A., Bergh J., Foekens J.A. (2007). Definition of clinically distinct molecular subtypes in estrogen receptor-positive breast carcinomas through genomic grade. J. Clin. Oncol..

[65] Bohmann K., Hennig G., Rogel U., Poremba C., Mueller B.M., Fritz P., Stoerkel S., Schaefer K.L. (2009). RNA extraction from archival formalin-fixed paraffin-embedded tissue: A comparison of manual, semiautomated, and fully automated purification methods. Clin. Chem..

[66] Hennig G., Gehrmann M., Stropp U., Brauch H., Fritz P., Eichelbaum M., Schwab M., Schroth W. (2010). Automated extraction of DNA and RNA from a single formalin-fixed paraffin-embedded tissue section for analysis of both single-nucleotide polymorphisms and mRNA expression. Clin. Chem..

[67] Muller B.M., Kronenwett R., Hennig G., Euting H., Weber K., Bohmann K., Weichert W., Altmann G., Roth C., Winzer K.J. (2011). Quantitative determination of estrogen receptor, progesterone receptor, and HER2 mRNA in formalin-fixed paraffin-embedded tissue—A new option for predictive biomarker assessment in breast cancer. Diagn. Mol. Pathol..

[68] Dubsky P., Brase J.C., Fisch K., Jakesz R., Singer C.F., Greil R., Dietze O., Weber K.E., Petry C., Kronenwett R. (2012). The EndoPredict score identifies late distant metastases in ER+/HER2− breast cancer patients. Cancer Res..

[69] Ein-Dor L., Kela I., Getz G., Givol D., Domany E. (2005). Outcome signature genes in breast cancer: Is there a unique set?. Bioinformatics.

[70] Dubsky P.C., Jakesz R., Mlineritsch B., Postlberger S., Samonigg H., Kwasny W., Tausch C., Stoger H., Haider K., Fitzal F. (2012). Tamoxifen and anastrozole as a sequencing strategy: A randomized controlled trial in postmenopausal patients with endocrine-responsive early breast cancer from the Austrian Breast and Colorectal Cancer Study Group. J. Clin. Oncol..

[71] Simon R.M., Paik S., Hayes D.F. (2009). Use of archived specimens in evaluation of prognostic and predictive biomarkers. J. Natl. Cancer Inst..

[72] Martin M., Brase J.C., Ruiz-Borrego M., Krappmann K., Munarriz B., Fisch K., Ruiz A., Weber K.E., Crespo C., Petry C. (2012). Prognostic performance of the EndoPredict score in node-positive chemotherapy-treated ER+/HER2− breast cancer patients: results from the GEICAM/9906 trial. Cancer Res..

[73] Brase J.C., Gehrmann M.C., Petry C., Weber K.E., Schmidt M., Kölbl H., Schroth W., Schwab M., Müller V., Jänicke F. (2011). The EndoPredict score is a response predictor for neoadjuvant chemotherapy in ER-positive, HER2-negative breast cancer. Cancer Res..

[74] Kronenwett R., Bohmann K., Prinzler J., Sinn B.V., Haufe F., Roth C., Averdick M., Ropers T., Windbergs C., Brase J.C. (2012). Decentral gene expression analysis: Analytical validation of the Endopredict genomic multianalyte breast cancer prognosis test. BMC Cancer.

[75] Denkert C., Kronenwett R., Schlake W., Bohmann K., Penzel R., Weber K.E., Hofler H., Lehmann U., Schirmacher P., Specht K. (2012). Decentral gene expression analysis for ER+/HER2− breast cancer: Results of a proficiency testing program for the EndoPredict assay. Virchows Arch..

[76] Noske A., Loibl S., Darb-Esfahani S., Roller M., Kronenwett R., Muller B.M., Steffen J., von Toerne C., Wirtz R., Baumann I. (2011). Comparison of different approaches for assessment of HER2 expression on protein and mRNA level: Prediction of chemotherapy response in the neoadjuvant GeparTrio trial (NCT00544765). Breast Cancer Res. Treat..

[77] Loibl S., Muller B.M., von Minckwitz G., Schwabe M., Roller M., Darb-Esfahani S., Ataseven B., du Bois A., Fissler-Eckhoff A., Gerber B. (2011). Androgen receptor expression in primary breast cancer and its predictive and prognostic value in patients treated with neoadjuvant chemotherapy. Breast Cancer Res. Treat..

[78] Muller B.M., Brase J.C., Haufe F., Weber K.E., Budzies J., Petry C., Prinzler J., Kronenwett R., Dietel M., Denkert C. (2012). Comparison of the RNA-based EndoPredict multigene test between core biopsies and corresponding surgical breast cancer sections. J. Clin. Pathol..

[79] Wöckel A., Kreienberg R. (2008). First revision of the German S3 guideline ‘diagnosis, Therapy, and Follow-Up of Breast Cancer’. Breast Care (Basel).

[80] Carlson R.W., Brown E., Burstein H.J., Gradishar W.J., Hudis C.A., Loprinzi C., Mamounas E.P., Perez E.A., Pritchard K., Ravdin P. (2006). NCCN task force report: Adjuvant therapy for breast cancer. J. Natl. Compr. Canc. Netw..

[81] Dubsky P., Filipits M., Jakesz R., Rudas M., Singer C.F., Greil R., Dietze O., Luisser I., Klug E., Sedivy R. (2013). EndoPredict improves the prognostic classification derived from common clinical guidelines in ER-positive, HER2-negative early breast cancer. Ann. Oncol..

[82] Muller B.M., Keil E., Lehmann A., Winzer K.J., Richter-Ehrenstein C., Prinzler J., Bangemann N., Reles A., Stadie S., Schoenegg W. (2013). The EndoPredict gene-expression assay in clinical practice—Performance and impact on clinical decisions. PLoS One.

[83] Ettl J., Große Lackmann K., Hapfelmeier A., Klein E., Paepke S., Petry C., Specht K., Höfler H., Kiechle M. Prospective Comparison of uPA/PAI-1 and Endopredict-Clin Score in ER-Positive, HER2-Negative Breast Cancer: Impact on Risk Stratification and Treatment Decisions. Proceeding of 2013 ASCO Annual Meeting.

[84] Blank P., Schwenkglenks M., Dubsky P., Filipits M., Gutzwiller F., Lux M.P., Brase J.C., Kronenwett R., Szucs T.D., Gnant M. (2013). Health economic analysis of guideline and gene expression signature-based risk stratification of distant recurrence in early breast cancer patients. Ann. Oncol..

[85] Weigelt B., Reis-Filho J.S., Swanton C. (2012). Genomic analyses to select patients for adjuvant chemotherapy: Trials and tribulations. Ann. Oncol..

[86] Nielsen T.O., Parker J.S., Leung S., Voduc D., Ebbert M., Vickery T., Davies S.R., Snider J., Stijleman I.J., Reed J. (2010). A comparison of PAM50 intrinsic subtyping with immunohistochemistry and clinical prognostic factors in tamoxifen-treated estrogen receptor-positive breast cancer. Clin. Cancer Res..

[87] Parker J.S., Mullins M., Cheang M.C., Leung S., Voduc D., Vickery T., Davies S., Fauron C., He X., Hu Z. (2009). Supervised risk predictor of breast cancer based on intrinsic subtypes. J. Clin. Oncol..

[88] Jankowitz R.C., Cooper K., Erlander M.G., Ma X.J., Kesty N.C., Li H., Chivukula M., Brufsky A. (2011). Prognostic utility of the breast cancer index and comparison to Adjuvant! Online in a clinical case series of early breast cancer. Breast Cancer Res..

[89] Jerevall P.L., Ma X.J., Li H., Salunga R., Kesty N.C., Erlander M.G., Sgroi D.C., Holmlund B., Skoog L., Fornander T. (2011). Prognostic utility of HOXB13:IL17BR and molecular grade index in early-stage breast cancer patients from the Stockholm trial. Br. J. Cancer.

[90] Varga Z., Sinn P., Fritzsche F., von Hochstetter A., Noske A., Schraml P., Tausch C., Trojan A., Moch H. (2013). Comparison of EndoPredict and oncotype DX test results in hormone receptor positive invasive breast cancer. PLoS One.

[91] Dowsett M., Sestak I., Lopez-Knowles E., Sidhu K., Dunbier A.K., Cowens J.W., Ferree S., Storhoff J., Schaper C., Cuzick J. (2013). Comparison of PAM50 risk of recurrence score with oncotype DX and IHC4 for predicting risk of distant recurrence after endocrine therapy. J. Clin. Oncol..

[92] Mamounas E.P., Tang G., Paik S., Baehner F.L., Liu Q., Jeong J.H., Kim S.R., Butler S.M., Jamshidian F., Cherbavaz D.B. (2012). Association between the 21-gene recurrence score (RS) and benefit from adjuvant paclitaxel (Pac) in node-positive (N+), ER-positive breast cancer patients (pts): Results from NSABP B-28. Cancer Res..

